# Evaluation of Transcutaneous Non-Invasive Blood Gas Analysis for Monitoring Gas Exchange in Pediatric Cardiac Surgical Patients Post Extubation

**DOI:** 10.21470/1678-9741-2024-0010

**Published:** 2025-03-24

**Authors:** Gaurav Pandey, Salman Pervaiz Butt, Arshad Ghori, Naveen G Singh

**Affiliations:** 1 Heart Vascular and Thoracic Institute, Cleveland Clinic, Abu Dhabi, United Arab Emirates.; 2 Department of Anesthesiology, Sri Jayadeva Institute of Cardiovascular Sciences and Research, Bengaluru, India.

**Keywords:** Child, Transcutaneous Blood Gas Monitoring, Carbon Dioxide, Oxygen, Calibration, Heart Failure, Cardiac Surgical Procedures, Hemodynamics

## Abstract

**Introduction:**

Pediatric cardiac surgery patients need close post-extubation monitoring for ventilation. Non-invasive transcutaneous partial pressure of oxygen (TcPO_2_) and transcutaneous partial pressure of carbon dioxide (TcPCO_2_) offer continuous insights and in improving care.

**Objective:**

To investigate the correlation of transcutaneous blood gases (TcPO_2_, TcPCO_2_) with arterial blood gases *i.e.* arterial partial pressure of oxygen (PaO_2_) and arterial partial pressure of carbon dioxide (PaCO_2_).

**Methods:**

We conducted a study on 30 pediatric post-cardiac surgery patients (four months to three years old) who were extubated and exhibited stable hemodynamics (inotropic score ≤ 5), normal sinus rhythm, and no respiratory or heart failure signs. Continuous transcutaneous and intermittent arterial blood gas monitoring started one hour after extubation, with recordings every 30 minutes for four hours. A single observer conducted probe calibration and data recording to minimize variability, while analysis of 240 paired samples included correlation coefficient, linear regression, Bland-Altman analysis, and Mountain plot.

**Results:**

The r-value between PaCO_2_ and TcPCO_2_ was 0.95, *r^2^*-value of 0.9060 (*P*<0.001). Bland-Altman showed a bias of 2.579, and 95% limits of agreement were -6.4 to 1.3. The *r*-value between PaO_2_ and TcPO_2_ was 0.8942, *r^2^*-value of 0.7996 (*P*<0.001); bias of 20.171 and 95% limit of agreement of -0.5 to 40.9. The Mountain plot revealed a median of 2.57 for PaCO_2_ *vs.* TcPCO2 and 20.17 for PaO_2_ *vs.* TcPO_2_.

**Conclusion:**

Transcutaneous carbon dioxide values are interchangeable with arterial PaCO_2_ in our population study, acting as a surrogate in postoperative pediatric cardiac surgery. Confirmation with arterial blood gases is needed if discrepancies occur.

## INTRODUCTION

Measurement of arterial blood gases is an integral part of monitoring of respiratory status of the patient. Extubated, postoperative pediatric cardiac surgical patients may become unstable rapidly if they are not monitored closely for hypercapnia and hypoxia. Arterial blood gas analysis remains the “gold standard” monitoring. Transcutaneous partial pressure of carbon dioxide (TcPCO_2_) monitoring has been done during neonatal transport^[1]^ and in pediatric patients (four years or older) receiving mechanical ventilation for respiratory failure^[2]^. The American Academy of Sleep Medicine recommends monitoring and reporting of hypoventilation in adults and pediatric population, and arterial partial pressure of carbon dioxide (PaCO_2_), TcPCO_2_, or end-tidal partial pressure of carbon dioxide (PCO_2_) can be used for detecting hypoventilation during a diagnostic study in both adults and children^[3]^. However, limited literature is available in extubated postoperative pediatric cardiac surgical patients. The purpose of this study was to observe the correlation of transcutaneous blood gases (transcutaneous partial pressure of oxygen [TcPO_2_], TcPCO_2_) with arterial blood gases in such patients.

## METHODS

This study was conducted at a tertiary care hospital, in the postoperative pediatric cardiac surgical intensive care unit, after obtaining informed consent from parents and was approved by the Institutional Ethical Committee of the Sri Jayadeva Institute of Cardiovascular Sciences and Research, Bangalore, India.

### Inclusion Criteria

Four-month-old to three-year-old pediatric patients who got extubated after cardiac surgery in postoperative pediatric cardiac surgical unit, had an arterial catheter in place with stable hemodynamic parameters, normal sinus rhythm, no residual shunts after cardiac surgery, no signs of cardiac and or respiratory failure, were normothermic, and had a low inotropic score of ≤ 5 were included in the study.

### Exclusion Criteria

Patients with unstable hemodynamic parameters, post-repair residual shunts, palliative procedures related to either single ventricle pathology or cyanosis, arrhythmias, signs of respiratory failure, low cardiac output, skin edema, and on high vasopressor support were excluded.

Transcutaneous monitoring (TCM) and arterial blood gas monitoring were started one hour after extubation and continued for four hours by using Draeger TcPO_2_ and TcPCO_2_ monitor. Transcutaneous probe calibration was done according to the manufacturer instructions (TINA TCM4, Radiometer, Copenhagen, Denmark). Before placement of transcutaneous probe, calibration was done with gas cylinder which was provided with the instrument. The probe was attached to the dry skin of the right or left upper chest. The working temperature of the probe was kept at 43°C, and the monitor site was changed every two hours to prevent any thermal injury to the patients. The probe was recalibrated before placing it to a new site. To minimize the inter-rater variability, probe calibration, placement, site change monitoring, and recording of data were done by a single observer, and the observer was unaware of arterial blood gas values which were taken every 30 minutes. Arterial blood gases (PCO_2_ and partial pressure of oxygen [PO_2_]) were recorded at an interval of 30 minutes, and transcutaneous gases (TcPCO_2_ and TcPO_2_) were recorded simultaneously, for a period of four hours. The transcutaneous gases’ values were displayed on Draeger Infinity delta XL monitor. A set of eight samples for arterial and transcutaneous gases were recorded for each patient.

### Statistical Analysis

Sample size was calculated based on a previous study^[4]^, considering correlation coefficient *r =* 0.9, alpha error = 0.05 with power = 80%. A total of 30 patients were included in the study. Pearson’s correlation was done to analyze the correlation coefficient r between transcutaneous gases and arterial gases. A linear regression *r^2^* and a Bland-Altman analysis^[5]^ were performed to compare the transcutaneous and arterial blood gas values. The bias, measured by Bland-Altman, represents the systemic error or variability between two techniques and is defined as the mean difference between values. Bland-Altman graphs were plotted for visual observation, and 95% confidence limit (limit of agreement) was estimated. In addition, folded cumulative distribution plot (Mountain plot) described by Krouwer and Mont A^[6]^ were also plotted.

A Mountain plot measures the difference of the value obtained by the standard method (arterial blood gases, *i.e.,* arterial partial pressure of oxygen [PaO_2_], PaCO_2_) and the method under investigation (transcutaneous gases, TcPCO_2_, TcPO_2_) on the x-axis and the percentile of differences on the y-axis. The resultant plot is inevitably a “mountain.” The benefits of the Mountain plot are that it is easier to find the central 95% of the data and easy to estimate percentile for large difference between methods. All the statistical analyses were done with MedCalc software version 12.2.1 (Ostend, Belgium).

## RESULTS

A total of 30 patients were included, from whom 240 paired samples between transcutaneous and arterial blood gases for both carbon dioxide (CO_2_) and oxygen were analyzed. There were 17 male and 13 female patients, their age varied from four months to three years, and their weight varied from 4.4 kg to 17 kg (mean ± standard deviation -10.08 ± 3.15). They underwent various types of intra-cardiac repair (Table 1).

**Table 1:** Number of postoperative patients who underwent various types of intracardiac repair.

PDA	ASD	VSD	ASD+VSD	TOF	TAPVC	PAPVC
1	4	11	3	7	2	2

ASD=atrial septal defect; PAPVC=partial anomalous pulmonary venous connection; PDA=patent ductus arteriosus; TOF=tetralogy of Fallot; VSD=ventricular septal defect

The TcPCO_2_ was higher than PaCO_2_ with mean difference of 2.6 ± 1.96 mmHg (PaCO_2_-TcPCO_2_). Pearson’s correlation coefficient *r*-value between TcPCO_2_ and PaCO_2_ was 0.9519, and linear regression analysis showed *r^2^*-value of 0.9060 (*P*<0.001) (Figure 1A). Bland-Altman showed a bias of 2.579, and 95% limit of agreement between PaCO_2_ and TcPCO_2_ was -6.4 to 1.3 (Figure 1B).

**Fig. 1A:**
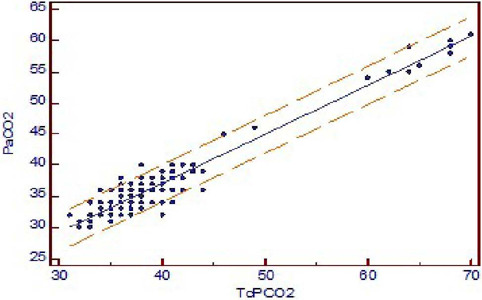
Linear regression analysis of arterial partial pressure of carbon dioxide (PaCO_2_) versus transcutaneous partial pressure of carbon dioxide (TcPCO_2_) value. The coefficient of determination r^2^ is 0.9060. y = 5.5259 + 0.7893 x.

**Fig. 1B:**
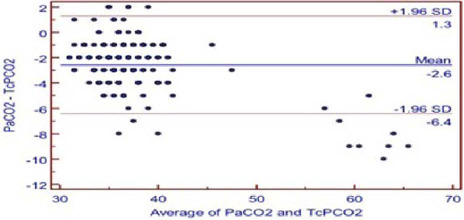
Bland-Altman analysis of agreement between arterial partial pressure of carbon dioxide (PaCO_2_) and transcutaneous partial pressure of carbon dioxide (TcPCO_2_). The difference (PaCO_2_ – TcPCO_2_) is plotted against the mean (PaCO_2_/2 + TcPCO_2_/2) for each value. The mean difference is -2.6 mmHg and limit of agreement is from -6.4 to 1.3. SD=standard deviation*.*

The mean difference between PaO_2_ and TCPO_2_ was 20.2 ± 1.96 mmHg (PaO_2_-TcPO_2_), and PaO_2_ was higher (Table 2). The *r*-value between PaO_2_ and TcPO_2_ was 0.8942, and linear regression analysis showed *r2*-value of 0.7996 (*P*<0.001) which indicates a strong correlation between PaO_2_ and TcPO_2_ (Figure 2A). Bland-Altman analysis of PaO_2_ and TcPO_2_ showed a bias of 20.171 and 95% limit of agreement of -0.5 to 40.9 (Figure 2B). The Mountain plot, which is generally used as complimentary to Bland-Altman plot, also showed similar results where the median PaCO_2_ and TcPCO_2_ was small (2.57) and showed small tail (Figure 1C). The median PaO_2_ and TcPO_2_ was large (20.17), with long tail (Figure 2C).

**Table 2:** Correlation coefficient, linear regression, and Bland-Altman analysis results between transcutaneous and arterial blood gases.

	PaCO_2_ – TcPCO_2_	PaO_2_ – TcPO_2_
*r*-value	0.951	0.894
*r^2^*-value	0.906	0.799
Bias	-2.6	20.2
Limit of agreement	-6.4 to 1.3	-0.5 to 40.9

PaCO_2_=arterial partial pressure of carbon dioxide; PaO_2_=arterial partial pressure of oxygen; TcPCO_2_=transcutaneous partial pressure of carbon dioxide; TcPO_2_=transcutaneous partial pressure of oxygen

**Fig. 1C:**
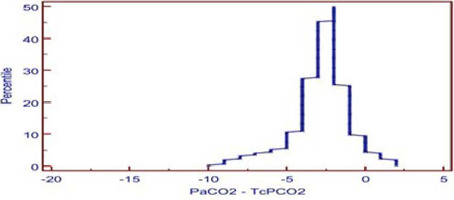
Mountain plot analysis between arterial and transcutaneous carbon dioxide. Values vary from 2.00 to -10.00. Median is -2.00. PaCO_2_=arterial partial pressure of carbon dioxide; TcPCO_2_=transcutaneous partial pressure of carbon dioxide.

**Fig. 2A:**
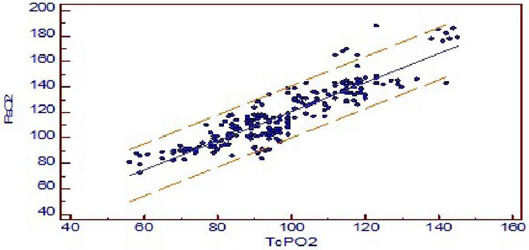
Linear regression analysis of arterial partial pressure of oxygen (PaO_2_) versus transcutaneous partial pressure of oxygen (TcPO_2_) value. The coefficient of determination r^2^ is 0.7996. y = 6.6815 + 1.1394 x.

**Fig. 2B:**
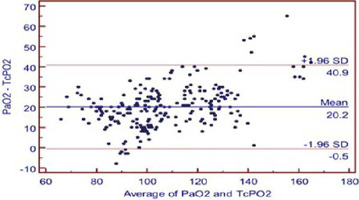
Bland-Altman analysis of agreement between arterial partial pressure of oxygen (PaO_2_) and transcutaneous partial pressure of oxygen (TcPO_2_). The difference (PaO_2_ – TcPO_2_) is plotted against the mean (PaO_2_/2 + TcPO_2_/2) for each value. The mean difference is 20.2 mmHg and limit of agreement is from -0.5 to 40.9. SD=standard deviation.

**Fig. 2C:**
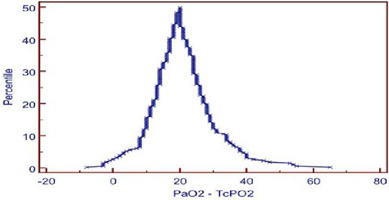
Mountain plot analysis between arterial and transcutaneous oxygen. Values vary from -8.00 to 65.00. Median is 20.00. PaO_2_=arterial partial pressure of oxygen; TcPO_2_=transcutaneous partial pressure of oxygen.

## DISCUSSION

After the analysis, we found that TcPCO_2_ was accurate and in close agreement with arterial PaCO_2_ in our postoperative pediatric cardiac surgical population study. Pearson’s correlation coefficient *r*-value is 0.9519 (*P*<0.001), which shows a strong positive correlation between PaCO_2_ and TcPCO_2_, and Bland-Altman analysis shows a bias of 2.6 and 95% confidence limit of agreement of -6.4 to 1.3 mmHg. The American Association of Respiratory Care (AARC) clinical practice guidelines has cited as clinically acceptable agreement between TcPCO_2_ and PaCO_2_ of ± 7.5 mmHg or 1 kPa for TCM of CO_2_ and oxygen^[7]^, in 2012. Mountain plot shows median of -2.0, which is very close to “0” and small tail, *i.e.,* less bias and more precise.

In recent studies, Karolina Weinmann et al.^[8]^ did continuous transcutaneous CO_2_ monitoring to avoid hypercapnia in complex catheter ablations under conscious sedation and found that it is feasible and precise with good correlation (*r*=0.60–0.87, *P*<0.005) to arterial blood gas CO_2_ analysis under conscious sedation and may contribute to additional safety.

Wang W et al.^[9]^ found, in pediatric laparoscopic surgery, that a close correlation (*r^2^*=0.70, *P*<0.01) was established between TcPCO_2_ and PaCO_2_. Compared to end-tidal CO_2_, transcutaneous CO_2_ can estimate PaCO_2_ accurately and could be used as an auxiliary monitoring indicator to optimize anesthesia management for laparoscopic surgery in children, however, it is not a substitute for end-tidal CO_2_.

Michel Toussaint et al.^[10]^ assessed the quality of peripheral oxygen saturation (or SpO_2_) and PCO_2_ recordings overnight via TCM in children with neurological conditions (out of 64 children, 42 used positive pressure respiratory support). They were able to make satisfactory clinical decisions in 91% of cases and concluded by saying that the quality of transcutaneous sensor recordings was acceptable, and clinical findings were deemed as satisfactory in the large majority of cases. Many studies have not only shown a strong correlation between the TcPCO_2_ and PaCO_2_, but also positively validated accuracy, high degree of interchangeability, and that sometimes and it may provide a better estimate of PaCO_2_ than end-tidal CO_2_ in pediatric population^[11,12,13,14,15]^.

In 2019, a systemic review and meta-analysis for precision and accuracy of transcutaneous CO_2_ monitoring by Aron Conway et al.^[16]^ has identified that there may be substantial differences between TcPCO_2_ and PaCO_2_ depending on the context in which this technology is used in clinical practice, but in their meta-analysis, the population limits of agreement between transcutaneous and arterial CO_2_ in pediatric intensive care unit and surgery was -5.1 to 4.4 mmHg, which was an acceptable agreement between TcCO_2_ and PaCO_2_ (± 7.5 mm Hg or 1 kPa)^[16]^.

Regarding transcutaneous oximetry (TcPO_2_), we found Pearson’s correlation coefficient *r*=0.894, and linear regression analysis showed *r^2^*-value of 0.7996 (*P*<0.001) which has a strong positive correlation between TcPO_2_ and PaO_2_, and when comparing with Bland-Altman analysis, it revealed a bias of 20.17 and wide limit of agreement. On Mountain plot analysis for PaO_2_ and TCPO_2_, the median between PaO_2_ and TcPO_2_ was 20.17, with long tail, indicating less precise and poor interchangeability.

Several studies have demonstrated that TCPO_2_ is not generally reliable^[15,17]^ and have also found to have a poor correlation, wide limit of agreement between TcPO_2_ and PaO_2_, and suggested that TCPO_2_ cannot be surrogate to PaO_2_.

TCM of gases really measures TcPO_2_ and TcPCO_2_, not PaO_2_ and PaCO_2_^[11]^, that could be the possible reason for the clinically acceptable difference between PaCO_2_ and TcPCO_2_ be ± 7.5 mmHg, as per AARC clinical guidelines^[7]^.

TcPO_2_ is an indirect measurement of PaO_2_ and does not reflect oxygen delivery or oxygen content. Complete assessment of oxygen delivery requires knowledge of hemoglobin saturation and cardiac output. TcPCO_2_ is an indirect measurement of PaCO_2_, but knowledge of delivery and content is not necessary to use TCM (TcPCO_2_) for assessment of ventilation.

TCM has traditionally been done by placing a heated sensor on the skin that increases the capillary blood flow and amount of oxygen diffusing to the sensor. Due to different diffusion rates, monitoring TcPCO_2_ can typically be achieved using lower temperatures of 38-42°C, which is not feasible for TcPO_2_, where temperature has to be kept at 43-44°C to achieve precise results^[18]^.

Epidermal and dermal cells consume oxygen and produce CO_2_, therefore TcPO_2_ is lower than PaCO_2_ and TcPCO_2_ is higher than PaCO_2_ irrespective of the sensor measuring temperature. This influence is minimized by applying a temperature-specific constant and a metabolic factor by the manufacturers^[19,20]^.

Arterial blood gas analysis is a gold standard technique but provides only momentary status. It is time consuming, and repeated sampling might lead to blood loss and anemia especially in neonates and pediatric postoperative cardiac surgical patients. Liebowitz RS et al.^[21]^ concluded that there is low but measurable morbidity associated with arterial catheterization as well. TCM is a continuous, noninvasive method, but transcutaneous probe placement requires expertise — improper placement, damaged membranes, trapped air bubbles, and inappropriate calibration techniques may affect its accuracy. Patient problems such as tissue hypoperfusion, the presence of edema, low cardiac output, and hypothermia may affect the measurements. Several studies have documented that vasoactive substances like dopamine, epinephrine, dobutamine, and norepinephrine did not affect TcPCO_2_/TcPO_2_ measurements^[7,22,23]^.

### Limitations

Limitations of the study were: this is a single-center, observational study, only patients with stable hemodynamic parameters without residual shunt and arrhythmias were studied, monitoring of the patients for a very limited time, and the fact that pH, base excess or deficit, serum electrolyte, hematocrit, and lactate cannot be obtained by this instrument. We need further larger randomized control studies to assess whether trends of changes in transcutaneous gases values can be reliable in post cardiac surgery pediatric patients.

## CONCLUSION

CO_2_ values obtained from TCM are interchangeable with those obtained from arterial blood gas analysis in our population study, unlike oxygen measurements which are not interchangeable. Hence TcPCO_2_ values can be used as a surrogate for arterial PaCO_2_ measurements in postoperative pediatric cardiac surgical patients. However, arterial blood gas analysis should be performed when transcutaneous gases do not appear consistent with clinical findings.

## Abbreviations, Acronyms & Symbols

AARC: American Association of Respiratory Care
ASD: Atrial septal defect
CO_2_: Carbon dioxide
PaCO_2_: Arterial partial pressure of carbon dioxide
PaO_2_: Arterial partial pressure of oxygen
PAPVC: Partial anomalous pulmonary venous connection
PCO_2_: Partial pressure of carbon dioxide
PDA: Patent ductus arteriosus
PO_2_: Partial pressure of oxygen
SD: Standard deviation
TCM: Transcutaneous monitoring
TcPCO_2_: Transcutaneous partial pressure of carbon dioxide
TcPO_2_: Transcutaneous partial pressure of oxygen
TOF: Tetralogy of Fallot
VSD: Ventricular septal defect

## References

[1] Tingay DG, Stewart MJ, Morley CJ (2005). Monitoring of end tidal carbon dioxide and transcutaneous carbon
dioxide during neonatal transport. Arch Dis Child Fetal Neonatal Ed.

[2] Tobias JD, Flanagan JF, Wheeler TJ, Garrett JS, Burney C (1994). Noninvasive monitoring of end-tidal CO_2_ via nasal
cannulas in spontaneously breathing children during the perioperative
period. Crit Care Med.

[3] Berry RB, Albertario CL, Harding SM, Lloyd RM, Plante DT, Quan SF, Troester MM, Vaughn BV, for the American Academy of Sleep Medicine (2018). The AASM Manual for the Scoring of Sleep and Associated Events: Rules,
Terminology and Technical Specifications, Version 2.5.

[4] Tobias JD, Wilson WR Jr, Meyer DJ (1999). Transcutaneous monitoring of carbon dioxide tension after
cardiothoracic surgery in infants and children. Anesth Analg.

[5] Bland JM, Altman DG (1986). Statistical methods for assessing agreement between two methods
of clinical measurement. Lancet.

[6] Krouwer JS, Monti KL (1995). A simple, graphical method to evaluate laboratory
assays. Eur J Clin Chem Clin Biochem.

[7] Restrepo RD, Hirst KR, Wittnebel L, Wettstein R (2012). AARC clinical practice guideline: transcutaneous monitoring of
carbon dioxide and oxygen: 2012. Respir Care.

[8] Weinmann K, Lenz A, Heudorfer R, Aktolga D, Rattka M, Bothner C (2021). Continuous transcutaneous carbon-dioxide monitoring to avoid
hypercapnia in complex catheter ablations under conscious
sedation. Int J Cardiol.

[9] Wang W, Zhao Z, Tian X, Ma X, Xu L, Shang G (2023). Noninvasive carbon dioxide monitoring in pediatric patients
undergoing laparoscopic surgery: transcutaneous vs. end-tidal
techniques. BMC Pediatr.

[10] Toussaint M, Buggenhoudt L, Pelc K (2021). Nocturnal transcutaneous blood gas measurements in a pediatric
neurologic population: a quality assessment. Dev Neurorehabil.

[11] Bhalla AK, Khemani RG, Hotz JC, Morzov RP, Newth CJ (2019). Accuracy of transcutaneous carbon dioxide levels in comparison to
arterial carbon dioxide levels in critically ill children. Respir Care.

[12] Urbano J, Cruzado V, López-Herce J, del Castillo J, Bellón JM, Carrillo A (2010). Accuracy of three transcutaneous carbon dioxide monitors in
critically ill children. Pediatr Pulmonol.

[13] Wilson J, Russo P, Russo J, Tobias JD (2005). Noninvasive monitoring of carbon dioxide in infants and children
with congenital heart disease: end-tidal versus transcutaneous
techniques. J Intensive Care Med.

[14] Berkenbosch JW, Lam J, Burd RS, Tobias JD (2001). Noninvasive monitoring of carbon dioxide during mechanical
ventilation in older children: end-tidal versus transcutaneous
techniques. Anesth Analg.

[15] Jung D, Lee S, Moon S, Lim S, Yoon Y, Choi S (2009). Non-invasive monitoring of oxygen and carbon dioxide tension:
accuracy of transcutaneous O2 and CO2 and Endtidal CO2. J Korean Soc Emerg Med.

[16] Conway A, Tipton E, Liu WH, Conway Z, Soalheira K, Sutherland J (2019). Accuracy and precision of transcutaneous carbon dioxide
monitoring: a systematic review and meta-analysis. Thorax.

[17] Green GE, Hassell KT, Mahutte CK (1987). Comparison of arterial blood gas with continuous intra-arterial
and transcutaneous PO2 sensors in adult critically ill
patients. Crit Care Med.

[18] Palmisano BW, Severinghaus JW (1990). Transcutaneous PCO2 and PO2: a multicenterstudy of
accuracy. J Clin Monit.

[19] Jakubowicz JF, Bai S, Matlock DN, Jones ML, Hu Z, Proffitt B (2018). Effect of transcutaneous electrode temperature on accuracy and
precision of carbon dioxide and oxygen measurements in the preterm
infants. Respir Care.

[20] Wimberley PD, Grønlund Pedersen K, Olsson J, Siggaard-Andersen O (1985). Transcutaneous carbon dioxide and oxygen tension measured at
different temperatures in healthy adults. Clin Chem.

[21] Liebowitz RS, Rippe JM, Rippe J, Irwin R, Albert J (1985). Intensive care medicine.

[22] Pellicer A, Valverde E, Elorza MD, Madero R, Gayá F, Quero J (2005). Cardiovascular support for low birth weight infants and cerebral
hemodynamics: a randomized, blinded, clinical trial. Pediatrics.

[23] Virkki A, Polo O, Saaresranta T, Laapotti-Salo A, Gyllenberg M, Aittokallio T (2008). Overnight features of transcutaneous carbon dioxide measurement
as predictors of metabolic status. Artif Intell Med.

